# GPR132 antagonists alleviate atherosclerosis by suppressing M1 polarization and subsequent VSMCs proliferation and migration as revealed by transcriptomic profiling

**DOI:** 10.1007/s00011-026-02342-2

**Published:** 2026-08-18

**Authors:** Yin Feng, Zheng Cao, Fan Zhang, Xinwei Zhou, Lilliane Aol, Li Su, Liqun He

**Affiliations:** 1 Department of Cardiology, Tongji Medical College, Traditional Chinese and Western Medicine Hospital of Wuhan, Huazhong University of Science and Technology, Wuhan, 430030 China; 2 Key Laboratory of Molecular Biophysics of Ministry of Education, College of Life Science and Technology, Huazhong University of Science and Technology, Wuhan, 430074 China

**Keywords:** GPR132, Macrophage, Polarization, Vascular smooth muscle cell, Atherosclerosis

## Abstract

**Background:**

Atherosclerosis (AS) is a chronic inflammatory disorder with substantial global prevalence, wherein macrophage polarization and vascular smooth muscle cells (VSMCs) phenotypic switching are critical pathogenic events. Although G protein-coupled receptor 132 (GPR132) is markedly expressed in macrophages, its specific role and mechanism in AS-associated macrophage polarization remain incompletely understood.

**Methods:**

Pharmacological antagonism of GPR132 was evaluated in high-fat diet-fed ApoE^−/−^ mice. Integrated single-cell RNA sequencing and transcriptome analyses from GEO datasets were performed to profile GPR132 expression in murine and human atherosclerotic plaques. In vitro, GPR132 overexpression and knockout macrophages were generated to assess M1/M2 polarization markers, and an indirect co‑culture system with VSMCs was established. Mechanistic investigations employed RNA‑sequencing and Western blotting, with pathway inhibition using SB203580 or GPR132 antagonists.

**Results:**

GPR132 antagonism attenuated atherosclerotic lesion progression and reduced pro‑inflammatory cytokine secretion without altering lipid profiles. GPR132 was enriched in pro‑inflammatory (M1‑like) macrophage subpopulations within plaques, which also upregulated VSMC‑promoting growth factors. GPR132 overexpression promoted M1 macrophage polarization and suppressed IL‑4‑induced M2 macrophage polarization, whereas knockout enhanced M2 and attenuated M1. Conditioned medium from GPR132‑overexpressing macrophages significantly enhanced VSMCs proliferation and migration. Mechanistically, GPR132 drove M1 macrophage polarization primarily through activation of the p38/MAPK signaling pathway, an effect reversible by SB203580 or GPR132 antagonists.

**Conclusions:**

GPR132 promotes pro‑inflammatory macrophage polarization via p38/MAPK signaling, and its antagonism mitigates atherosclerotic progression by suppressing inflammatory microenvironments and VSMCs proliferation/migration, highlighting GPR132 as a potential therapeutic target for AS.

**Supplementary Information:**

The online version contains supplementary material available at 10.1007/s00011-026-02342-2.

## Introduction

Atherosclerosis (AS) is the most prevalent cardiovascular disease worldwide and represents the primary pathophysiological basis of coronary heart disease and cerebral infarction [1, 2]. Despite improvements in prevention and treatment, AS remains a leading cause of global mortality [3, 4]. The progression of AS is driven by chronic vascular inflammation and the aberrant proliferation and migration of vascular smooth muscle cells (VSMCs) [5, 6]. Under pathological conditions, VSMCs switch from a contractile to a secretory phenotype, acquiring high proliferative and migratory capacity that contributes to luminal narrowing [7, 8]. Inflammation is a key driver of VSMCs activation, with macrophage polarization playing a central role in promoting chronic vascular inflammation and VSMCs dysfunction [9–11].

Macrophages, as key mediators of the inflammatory response, are deeply involved in the pathogenesis and progression of AS [12, 13]. The role of macrophages in atherosclerosis is closely related to the phenotypes resulting from their polarization [14]. M1 macrophages predominantly accumulate in unstable plaques, where their presence accelerates plaque expansion and increases vulnerability. In contrast, M2 macrophages secrete anti-inflammatory cytokines including interleukin-10 (IL-10) and transforming growth factor-beta (TGF-β), which ameliorate AS progression and promote plaque stability [15, 16]. Importantly, inhibition of M1 macrophage activity significantly suppresses VSMCs activation, thereby reducing the risk of plaque rupture [17, 18]. Consequently, targeting macrophage polarization represents a promising therapeutic strategy for AS.

GPR132 (also known as G2A) is a G protein-coupled receptor widely expressed across various tissues, with particularly high levels in immune cells [19]. In inflammatory settings, GPR132 deficiency attenuates inflammation by reducing immune cell infiltration and cytokine production at injury sites [20]. Additionally, lactate promotes macrophage senescence via the GPR132–Src pathway, accelerating AS progression in diabetic mouse models [21]. These observations suggest that GPR132 plays a broad role in systemic inflammatory responses and may contribute to AS pathogenesis, although its precise mechanisms in AS remain poorly defined.

In the present study, it is demonstrated GPR132 drives M1 macrophage polarization through the p38/MAPK pathway, thereby enhancing VSMCs proliferation and migration. Pharmacological inhibition of GPR132 reduces atherosclerotic burden in ApoE^−/−^ mice, revealing GPR132 as a candidate therapeutic target in AS.

## Results

### GPR132 antagonist administration attenuated the progress of AS in ApoE^−/−^ mice

**Fig. 1:**
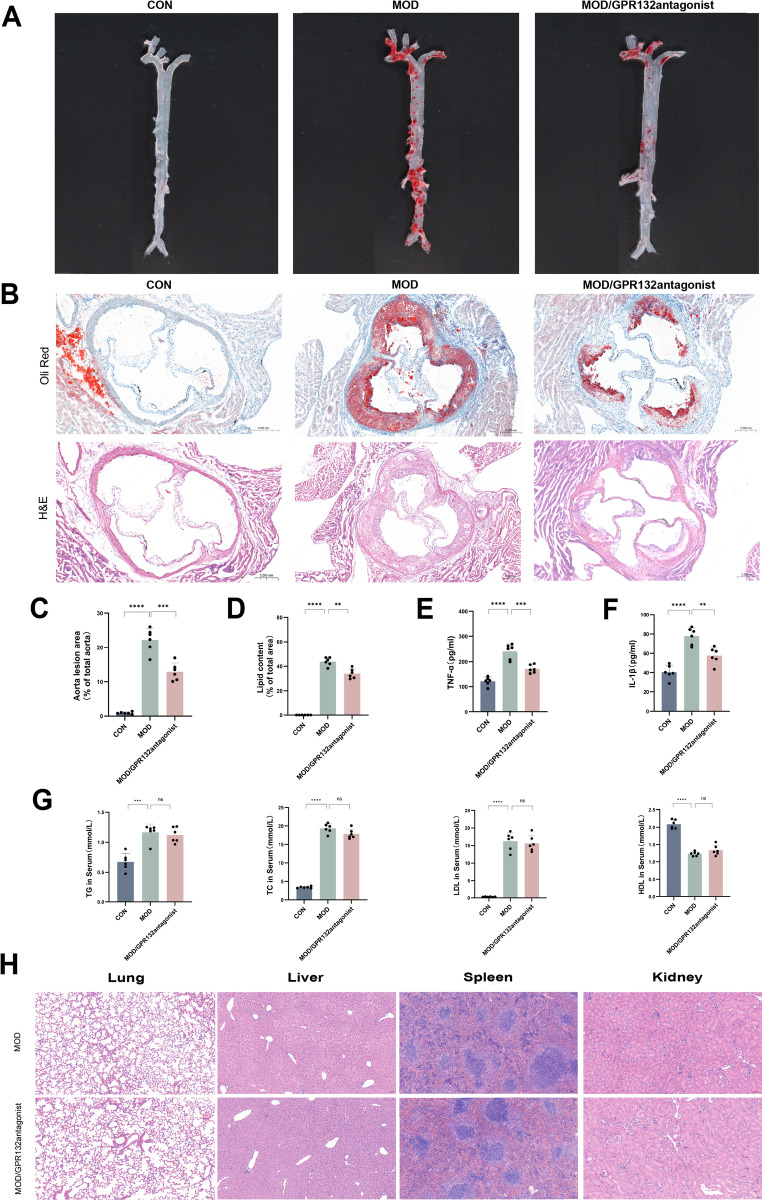
Intraperitoneal injection of GPR132 antagonists can slow the progression of AS and improve the inflammatory response in ApoE^−/−^ mice. Animals were divided into CON, MOD, and MOD/GPR132 Antagonist groups. At the end of the experiment, tissues were collected and stained with H&E and Oil Red O, serum was collected for analysis of IL-1β and TNF-α levels, and lipid levels (TC, TG, LDL, and HDL) were measured. **A** Oil red O staining of the entire aortic surface in mice. *n* = 6. **B** Representative H&E staining and oil red O staining of atherosclerotic lesions in the aortic sinus of each group. *n* = 6. Scale bar, 200 μm. **C** Quantitative analysis of aortic lesion areas in mice. *n* = 6. **D** Quantitative analysis of lipid content in Oil Red O-stained atherosclerotic plaques. *n* = 6. **E** TNF-α levels in serum. *n* = 6. **F** Il-1β levels in serum. *n* = 6. **G** Lipid levels (TC, TG, LDL, and HDL) in serum. *n* = 6. **H** H&E staining showing pathological changes in key organs (lung, liver, spleen, and kidney). *n* = 6, scale bar = 200 μm. Data are presented as mean ± SD. For in vivo experiments (**A**–**H**), *n* = 6 biologically independent mice per group. All ELISA measurements were conducted in triplicate technical replicates. Statistical analysis was performed using One-way ANOVA followed by Tukey’s test for (**C**–**G**). *p < 0.05, **p < 0.01, ***p < 0.001, ****p < 0.0001, ns: not significant

ApoE^−/−^ mice represent the most widely employed animal model for assessing anti-atherosclerotic therapeutics. Atherosclerotic plaque formation was induced by feeding the mice a high-fat diet (HFD) for 12 weeks. To investigate whether pharmacological inhibition of GPR132 attenuates atherosclerosis progression, ApoE^−/−^ mice received intraperitoneal injections of a GPR132 antagonist (2 mg/kg) or vehicle from week 8 to week 12 of HFD feeding. Throughout the experimental period, no significant differences in body weight or food intake were observed among the groups (Supplementary Fig. 1A). Compared with the high-fat diet-induced model (MOD) group, the MOD group treated with the GPR132 antagonist (MOD/GPR132 antagonist) displayed a notable reduction in atherosclerotic plaque burden in both the en face aorta and the aortic root (Fig. 1A). Consistent with this, Oil Red O staining of the aortic root sections revealed that the plaque lesion area was significantly larger in the MOD group than in the normal chow-fed control (CON) group. Conversely, administration of the GPR132 antagonist significantly decreased the plaque area compared to the MOD group (Fig. 1B). To evaluate the impact of GPR132 antagonist on the inflammatory response in ApoE^−/−^ mice, serum levels of key inflammatory cytokines were measured. The results demonstrated that the MOD/GPR132 antagonist group exhibited significantly lower concentrations of the prototypical pro-inflammatory cytokines TNF-α and IL-1β relative to the MOD group (Fig. 1E and F). However, the GPR132 antagonist did not improve lipid levels in mice. No significant differences were detected in total cholesterol (TC), triglycerides (TG), low-density lipoprotein (LDL), or high-density lipoprotein (HDL) between the MOD and MOD/GPR132 antagonist groups (Fig. 1G), suggesting that the attenuation of atherosclerosis by the GPR132 antagonist is independent of lipid metabolism (Fig. 1G). Notably, to assess potential off-target toxicity, histological examination via H&E staining was performed on major organs. The results indicated no apparent immune-mediated damage or significant pathological alterations in the lungs, livers, spleens, or kidneys of mice treated with the GPR132 antagonist (Fig. 1H).

### Expression of GPR132 is up-regulated in M1 macrophages

**Fig. 2:**
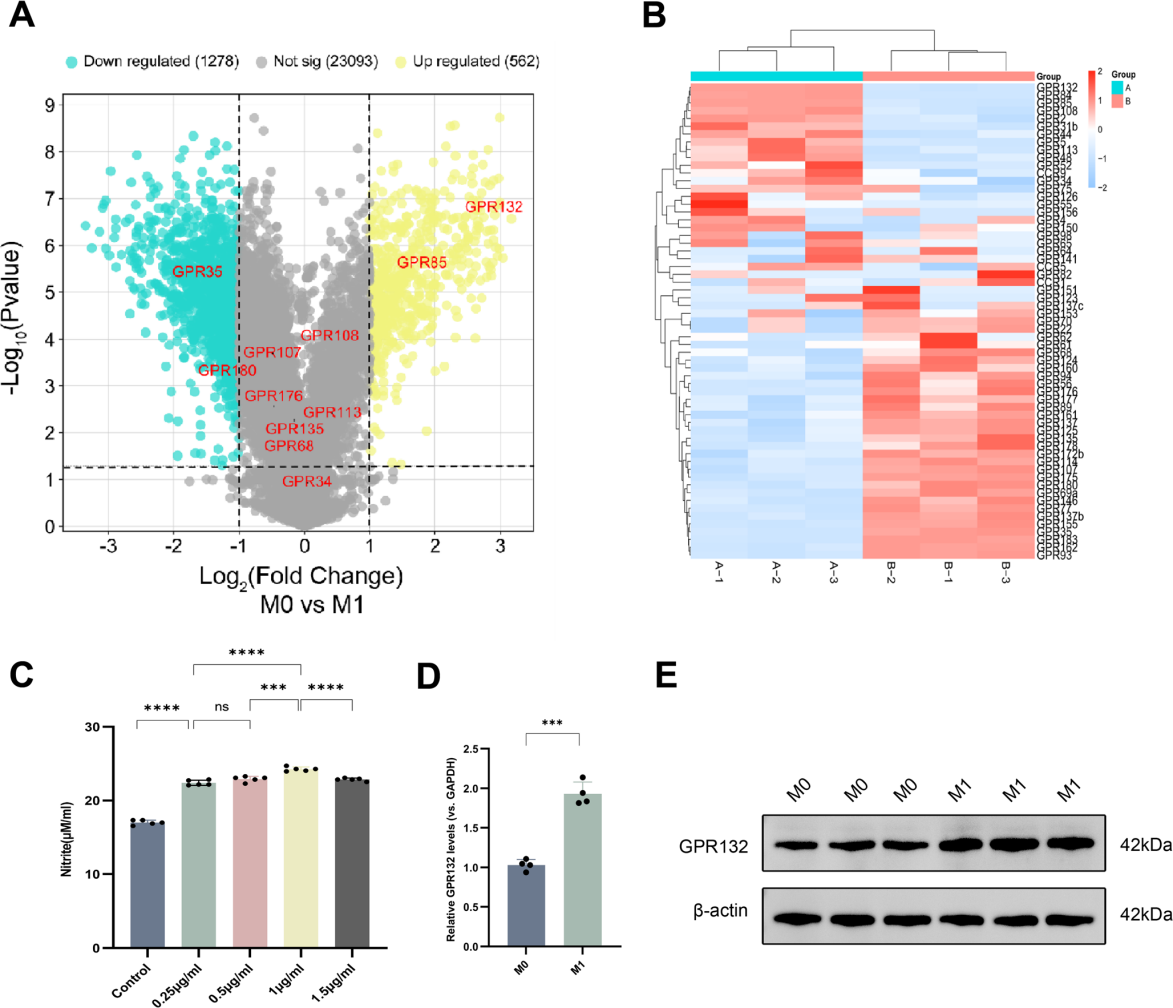
Differential expression analysis of GPR132 in M0 and M1 macrophages. **A** Volcano plot of DEGs between M0 and M1 macrophages. Red and blue dots represent upregulated (*n* = 562) and downregulated (*n* = 1278) genes, respectively, based on the thresholds of |Log₂(Fold Change)| > 1 and *p* value < 0.05 (dashed lines). **B** Heatmap of GPCR family genes expression in M0 (Control) and M1 macrophages. **C** RAW264.7 cells were treated with varying concentrations of LPS (0, 0.25, 0.5, 1 and 1.5 µg/mL) for 24 h. Nitric oxide (NO) production in the culture supernatant was measured using the Griess assay. *n* = 5. **D** RT-qPCR analysis of GPR132 mRNA levels in M0 versus M1 macrophages. *n* = 4. **E** Western blot showing the protein expression of GPR132 in M0 and M1 macrophages, with β‑actin as loading control. Data are presented as mean ± SD. For in vitro experiments in this study were all repeated for at least three times. Within each independent experiment, four technical replicates (wells) were set for qPCR analysis and Nitric Oxide assay; the mean values of these technical replicates were used for statistical calculations. Statistical analysis was performed using One-way ANOVA followed by Tukey’s test for (**C**). Statistical analysis was determined using Student’s t test for (**D**). *p < 0.05, **p < 0.01, ***p < 0.001, ****p < 0.0001, ns: not significant

First, a combination of three transcriptomic datasets from the GEO database (GSE183077, GSE230444, GSE283748), which documented gene expression profiles of RAW264.7 cells (a murine macrophage cell line) in the M0 state and during their polarization to the M1 phenotype, was utilized. Differential gene expression analysis (DEGs) was performed on these datasets and identified 562 upregulated genes and 1278 downregulated genes during the transition from M0 to M1 macrophages (Fig. 2A). Heatmaps showed the up- and down-regulated genes of GPCR family molecules during M1 polarization of macrophages (Fig. 2B). These results indicated that GPR132, a member of the GPCR family, was significantly expressed in M1 macrophages. To validate the high expression of GPR132 in M1 macrophages, M1 polarization was induced in macrophages using LPS. NO is a hallmark marker of M1 macrophages, and LPS stimulation of RAW264.7 cells increases NO production [22]. NO generation peaked at 1 µg/ml LPS stimulation and subsequently decreased with increasing LPS concentrations (Fig. 2C) (NO standard curve in Supplementary Fig. 2A). Accordingly, GPR132 expression was assessed 24 h after treatment with 1 µg/mL LPS. Results demonstrated that GPR132 expression was upregulated in M1 macrophages (Fig. 2D). To further confirm this at the protein level, Western blotting was performed under the same conditions. Compared with M0 macrophages, the expression of the GPR132 protein was significantly elevated in LPS-induced M1 macrophages, consistent with the previous bioinformatics analysis findings (Fig. 2E).

### Single-cell RNA sequencing reveals predominant GPR132 + macrophages correlate with inflammatory cytokines and pro-VSMC growth factors

**Fig. 3:**
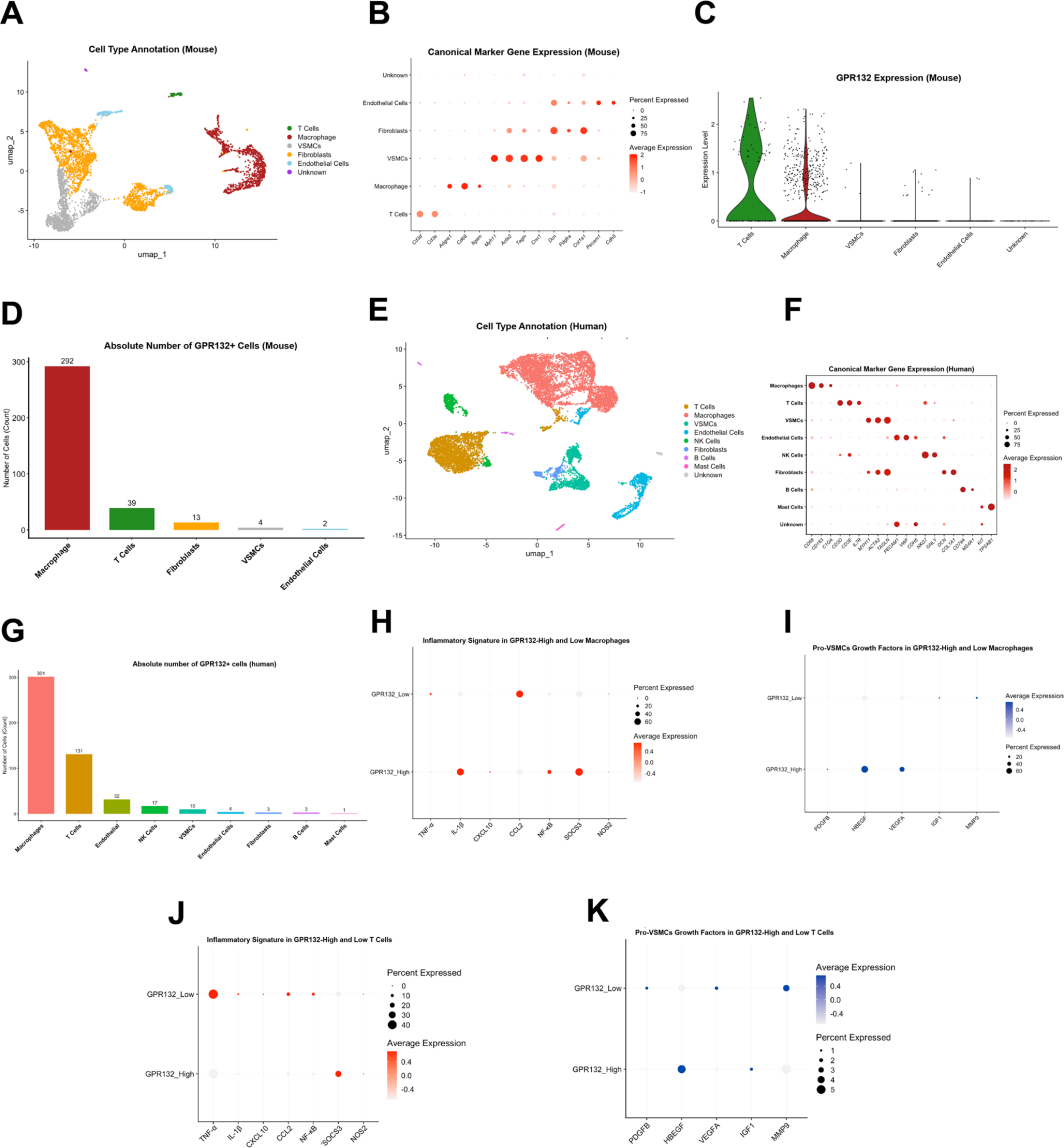
Single-cell RNA sequencing identifies GPR132 as a macrophage-specific driver of inflammation and VSMCs proliferation and migration in AS. **A** UMAP visualization of scRNA-seq data from ApoE^−/−^ mouse plaques, annotating major cell types. **B** Dot plot of canonical marker genes validating mouse cell clusters. **C** Violin plot illustrating the expression distribution of GPR132, showing enrichment in T cells and macrophages. **D** Bar plot quantifying the absolute number of GPR132-positive cells. Macrophages (*n* = 292) represent the primary Gpr132-expressing population. **E** UMAP visualization of human plaque clusters, identifying macrophages, T cells, VSMCs, and endothelial cells. **F** Dot plot validating human cell annotations with lineage-specific markers. **G** Bar plot quantifying the absolute number of GPR132-positive cells. Macrophages (*n* = 301) represent the primary GPR132-expressing population. **H** Dot plot comparing inflammatory markers (TNF-α, IL-1β, NF-κB, SOCS3) between GPR132-high and GPR132-low macrophages. **I** Dot plot showing specific upregulation of paracrine growth factors (HBEGF, VEGFA) in GPR132-high macrophages. **J** Dot plot comparing inflammatory markers between GPR132-High and GPR132-Low T cells. Note the absence of significant upregulation in M1 markers. **K** Dot plot of paracrine growth factors in T cells. The lack of VEGFA and HBEGF upregulation confirms that VSMCs proliferation and migration signals are macrophage-specific

To delineate the cellular context of GPR132 in AS, single-cell RNA sequencing (scRNA-seq) data from ApoE^−/−^ mouse plaques (GSE155513) was first analyzed. Unsupervised clustering identified distinct cell populations (Fig. 3A and B). Quantitative analysis revealed that GPR132 expression was predominantly enriched in macrophages and T cells (Fig. 3C). Notably, macrophages constituted the largest population of GPR132-positive cells (Fig. 3D). The analysis was next extended to human atherosclerotic plaques (GSE159677) to determine whether GPR132 is similarly enriched in the macrophage population and whether GPR132-High macrophages upregulate specific factors promoting VSMCs proliferation and migration. Conserved cell clusters were successfully annotated (Fig. 3E and F). Consistent with murine data, quantitative profiling identified macrophages as the dominant GPR132-positive population (301 cells), followed by T cells (131 cells) (Fig. 3G). The functional consequences of GPR132 signaling were then investigated. Differential expression analysis in macrophages revealed that the GPR132-high subpopulation exhibits a potent pro-inflammatory (M1-like) signature, characterized by significant upregulation of IL1β, NF-κB, and SOCS3 (Fig. 3H). Moreover, GPR132-High macrophages showed specific upregulation of HBEGF and VEGFA (Fig. 3I), suggesting they promote VSMCs proliferation and migration through paracrine signaling. Crucially, despite expressing GPR132, T cells lacked the pathogenic features observed in macrophages. They showed no significant upregulation of inflammatory cytokines (Fig. 3J) or paracrine growth factors like VEGFA and HBEGF (Fig. 3K). This confirms that the GPR132-mediated pro-inflammatory and paracrine phenotype is restricted to the macrophage lineage.

### GPR132 promotes macrophage M1 polarization and inhibits macrophage M2 polarization

**Fig. 4:**
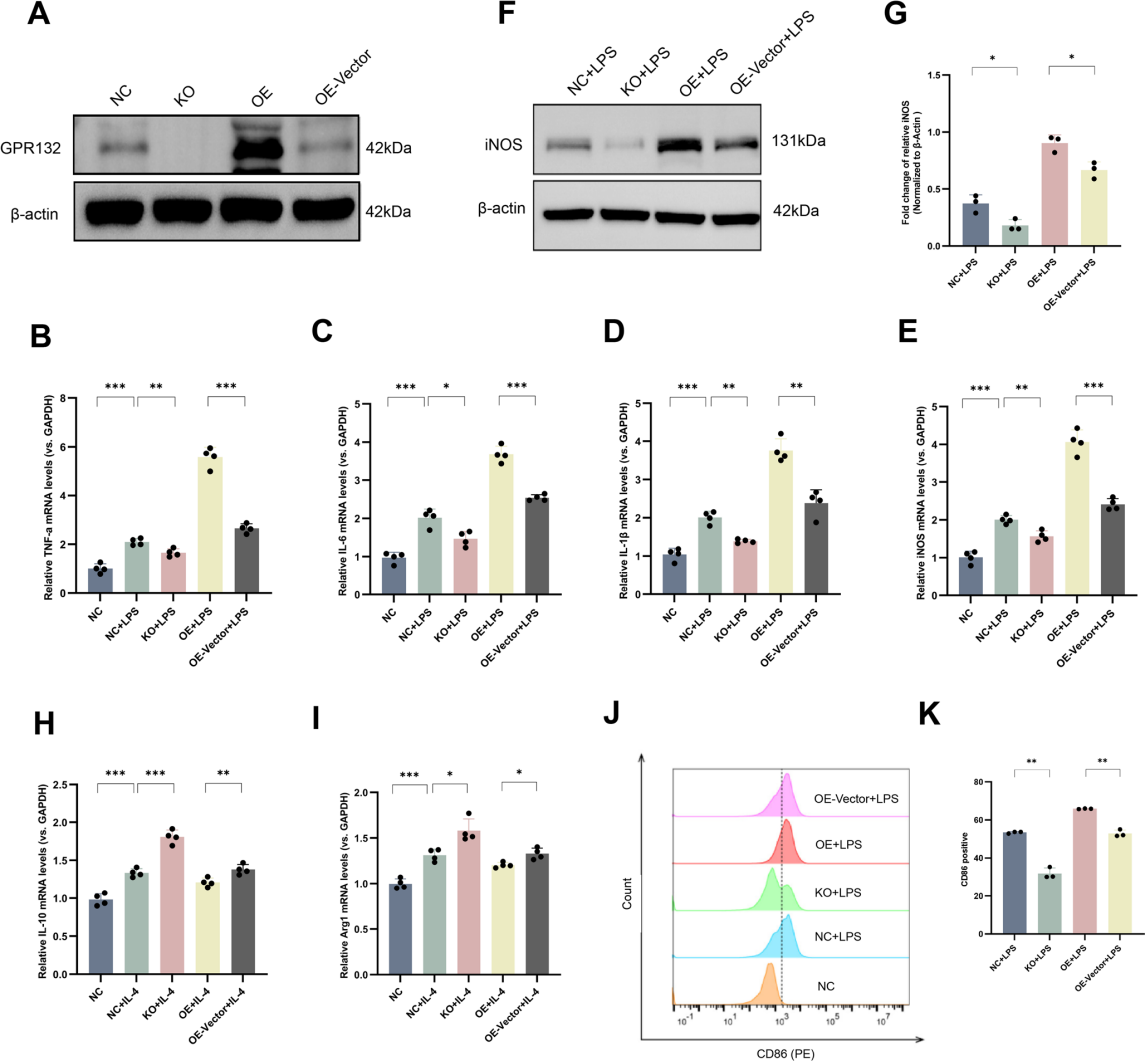
GPR132 promotes macrophage M1 polarization. **A** Western blot analysis confirming successful GPR132-OE and GPR132-KO in macrophages. **B**–**E** RT-qPCR analysis of M1 macrophage-associated gene expression markers, including TNF-α, IL-6, IL-1β, and iNOS. *n* = 4. **F**, **G** Western blot analysis of the protein levels of iNOS, a marker specific to M1 macrophages.*n* = 3. **H**–**I** RT-qPCR analysis of M2 macrophage-associated gene expression markers, including IL-10 and Arg1. *n* = 4. **J**, **K** Flow cytometry was used to evaluate the surface expression and quantitative analysis of M1 macrophage marker CD86. *n* = 3. Data are presented as mean ± SD. For in vitro experiments in this study were all repeated for at least three times. Within each independent experiment, four technical replicates (wells) were set for qPCR analysis; the mean values of these technical replicates were used for statistical calculations. Statistical analysis was performed using One-way ANOVA followed by Tukey’s test for (**B**–**G**), (**H** and **I**) and (**K**). *p < 0.05, **p < 0.01, ***p < 0.001, ****p < 0.0001

To further investigate the functional role of GPR132 in macrophage polarization, GPR132-knockout (KO) macrophages were developed via CRISPR-Cas9 gene editing. Additionally, using lentiviral transduction, macrophages overexpressing GPR132 (OE) and corresponding empty vector controls (OE-Vector) were established. Successful modulation of GPR132 protein levels was confirmed by Western blot (Fig. 4A). To assess the impact of GPR132 on M1 polarization, RAW264.7 cells were stimulated with LPS (1 µg/ml) and IL-4 (20 ng/ml) for 24 h, followed by detection of expression levels of classical M1 markers (TNF-α, IL-6, IL-1β, and iNOS) and M2 markers (IL-10 and Arg1). Quantitative PCR revealed that LPS and IL-4 treatment significantly increased mRNA expression of M1 and M2 markers in RAW264.7 cells, confirming successful induction of M1 and M2 polarization. GPR132-OE significantly upregulated the mRNA levels of TNF-α, IL-6, IL-1β, and iNOS upon LPS stimulation (Figs. 4B–E), indicating enhanced M1 polarization. Conversely, GPR132-KO markedly attenuated the induction of these M1-associated markers in response to LPS (Fig. 4B–E). Consistent with the mRNA data, iNOS protein expression followed a similar pattern, as shown by Western blot and its densitometric quantification (Fig. 4F–G). In the context of IL-4 stimulation, GPR132-KO promoted M2 polarization, whereas GPR132-OE suppressed this process (Fig. 4H and I). Specifically, compared to the NC + LPS control group, GPR132-KO reduced the mRNA expression of TNF-α, IL-6, IL-1β, and iNOS by approximately 21%, 27%, 31%, and 22%, respectively, while increasing IL-10 and Arg1 expression by approximately 35% and 20%, respectively, upon IL-4 stimulation. Moreover, relative to the OE-Vector + LPS control, GPR132-OE elevated TNF-α, IL-6, IL-1β, and iNOS levels by approximately 110%, 45%, 58%, and 77%, respectively, but decreased IL-10 and Arg1 expression by approximately 12% and 9%, respectively, under IL-4 treatment. In addition, this result was further demonstrated by flow cytometry. Among them, the expression level of CD86, a marker of M1 macrophages, was significantly higher in the LPS group compared with the blank group, indicating successful induction of M1 polarization. Notably, GPR132-OE further elevated CD86 levels, whereas GPR132-KO substantially reduced its expression (Fig. 4J and K). These findings demonstrate that GPR132 exerts opposing regulatory effects on macrophage polarization: its overexpression promotes M1 while inhibiting M2 polarization, whereas its knockout enhances M2 but attenuates M1 polarization.

### GPR132 overexpressing M1 macrophage supernatant promotes proliferation and migration of VSMCs

**Fig. 5:**
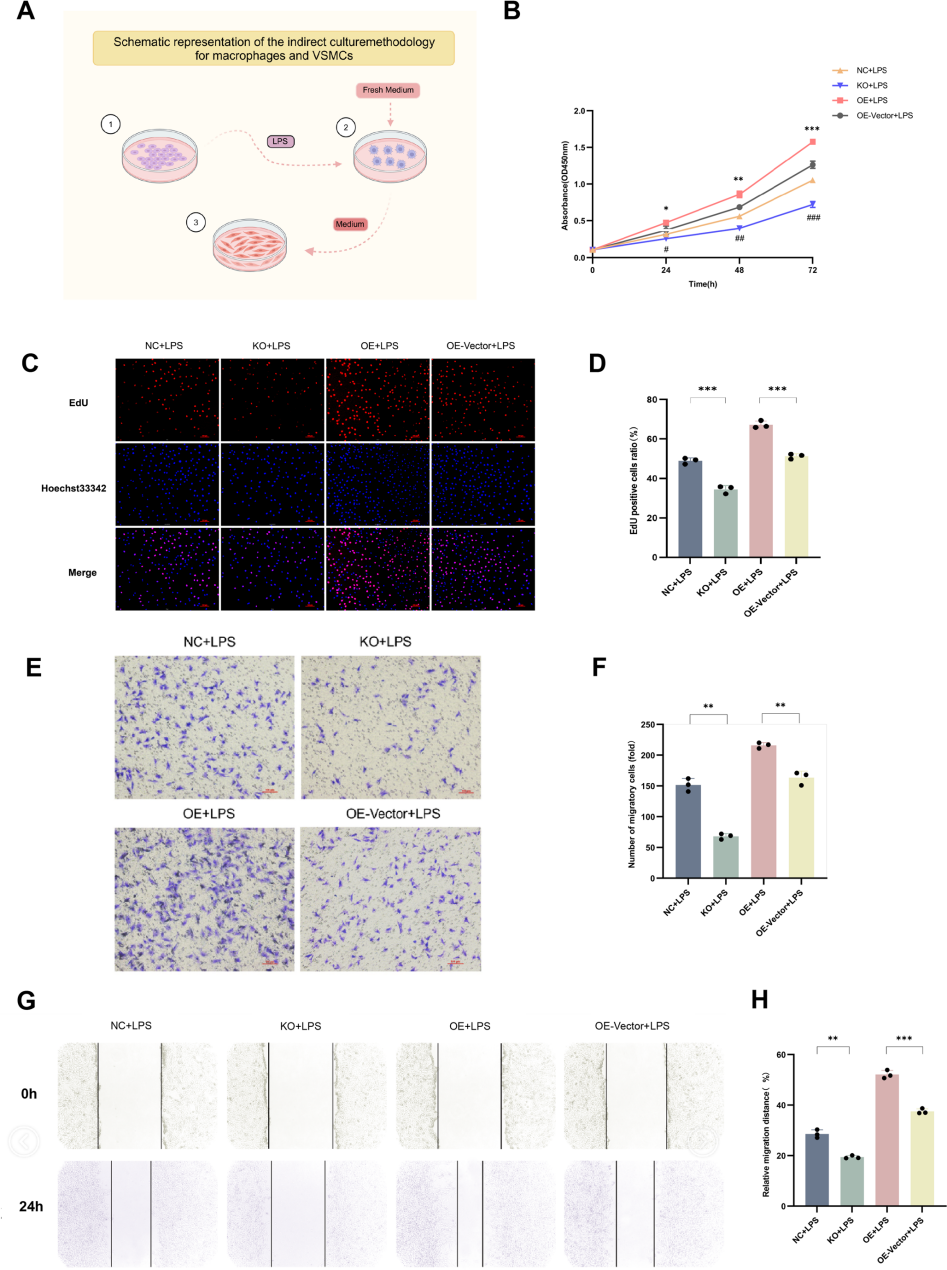
Effect of GPR132-OE and GPR132-KO in M1 macrophages supernatant on proliferation and migration of VSMCs. **A** Flow chart of indirect co-culture of macrophages with VSMCs. **B** Cell proliferation ability of VSMCs after co-culture was detected using CCK8. *n* = 4. **C**, **D** Percentage and quantitative analysis of EdU-positive cells. *n* = 3. Scale bar = 100 μm. **E**, **F** Transwell assay to detect the migration ability of VSMCs after co-culture. *n* = 3. Scale bar = 100 μm. **G**, **H** VSMCs migration distance was detected using Wound healing assay. Bar graphs show the difference in wound area reduction between different groups. *n* = 3. Scale bar = 100 μm. Data are presented as mean ± SD. For in vitro experiments in this study were all repeated for at least three times. Within each independent experiment, four technical replicates (wells) were set for the Transwell migration, CCK-8 proliferation, and Edu proliferation assays; and three technical replicates (wells) were set for the scratch assay; the mean values of these technical replicates were used for statistical calculations. Statistical analysis was performed using One-way ANOVA followed by Tukey’s test for (**D**), (**F**) and (**H**). *p < 0.05, **p < 0.01, ***p < 0.001

To investigate the effect of GPR132 in macrophages on the biological functions of VSMCs, in vitro co-culture experiments were performed. The specific procedure is illustrated in Fig. 5A. In the experiment, LPS was used to induce GPR132-OE and GPR132-KO macrophages to polarize to the M1 type. After 24 h, the culture supernatant of these macrophages and fresh culture medium were added to VSMCs in equal proportions to detect their effects on VSMCs proliferation and migration. CCK8 assay confirmed that GPR132-OE macrophages supernatant significantly promoted the proliferation of VSMCs, whereas GPR132-KO significantly inhibited the proliferative activity of VSMCs in macrophage supernatants (Fig. 5B). In addition, EdU proliferation assay demonstrated that GPR132-OE macrophages supernatant significantly increased the proportion of EdU-positive cells in VSMCs, whereas GPR132-KO resulted in a significant decrease in EdU-positive cells (Fig. 5C and D). VSMCs migratory capacity was next evaluated using wound healing and Transwell assays. The scratch wound closure rate was substantially enhanced in VSMCs treated with GPR132-OE macrophage-conditioned medium, but was suppressed in the GPR132-KO group (Fig. 5E and F). Consistent with this, Transwell migration assays showed a significant increase in the number of migrated VSMCs following exposure to GPR132-OE macrophage supernatant, whereas GPR132-KO supernatant strongly inhibited cell migration (Fig. 5G and H). Thus, the results suggest that GPR132-OE M1 macrophage supernatant promotes the proliferation and migration of VSMCs, whereas GPR132-KO reduces this process.

### Differential expression analysis of GPR132 overexpression in M1 macrophages

**Fig. 6:**
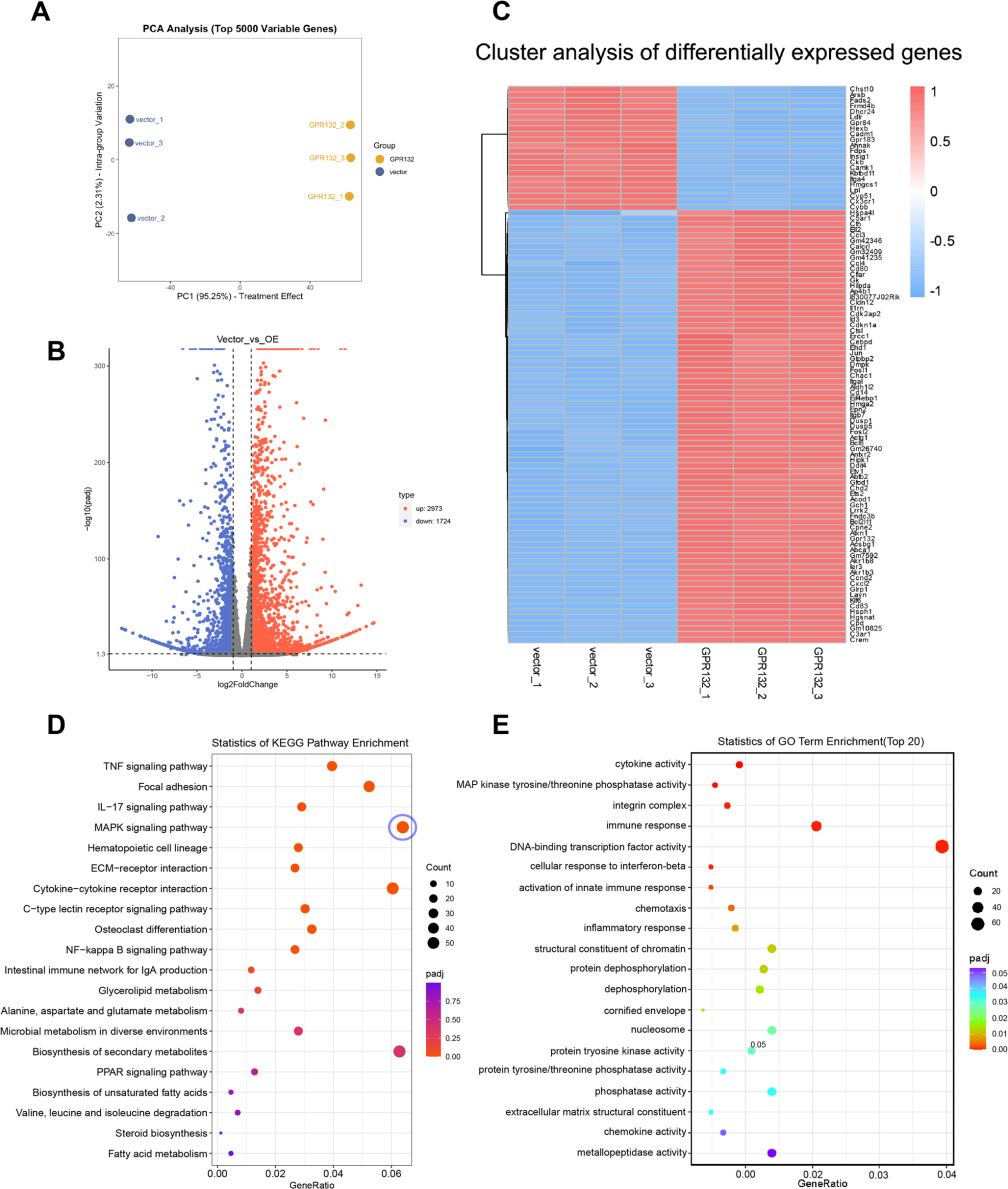
Differential expression analysis of GPR132-OE in M1 macrophages. **A** Principal component analysis in the OE group and OE-vector group. To assess the overall impact of GPR132-OE on the cellular transcriptome,analysis was performed using the top 5000 genes with the highest variance. **B** Volcano of DEGs in the two groups. **C** Heatmap of DEG in the two groups, analyzing the top 100 genes. **D**, **E** KEGG pathway enrichment analysis; GO enrichment analysis

To explore the potential mechanism of GPR132 on macrophage M1 polarization, RNA-seq analysis was performed on M1-type macrophages from GPR132-OE and GPR132-OE-Vector. Principal component analysis (PCA) on the top 5000 most variable genes revealed distinct clustering between the Vector and OE groups, indicating significant intergroup differences (Fig. 6A). DEG analysis was performed on the gene dataset and identified 2973 up-regulated genes and 1724 down-regulated genes (Fig. 6B). Meanwhile, the 100 genes with the most significant expression differences were screened for heatmapping, showing the up- and down-regulated genes in the Vector group versus the OE group (Fig. 6C). Seventy-six GO terms and 47 KEGG pathways were explored by GO and KEGG enrichment analysis with a threshold of *P* < 0.05. The results showed that DEGs were highly enriched in inflammation-related pathways between the GPR132-OE and GPR132-OE-Vector groups. Among them, they were mainly involved in MAPK signaling pathway, TNF signaling pathway, NF-κB, IL-17 signaling pathway and cytokine-cytokine receptor interactions (Fig. 6D and E).

### GPR132 promotes M1 macrophage polarization through the p38/MAPK pathway, thereby enhancing the proliferation and migration of VSMCs

**Fig. 7:**
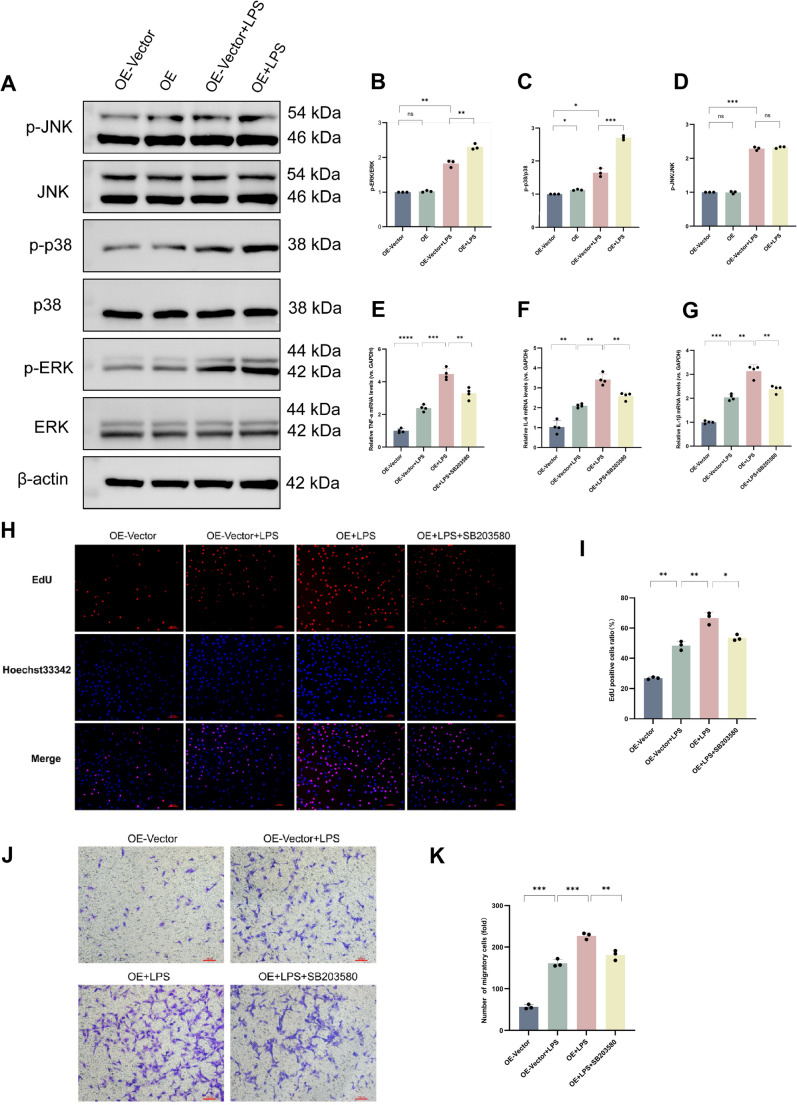
GPR132 promotes M1 macrophage polarization through the p38/MAPK pathway, thereby enhancing the proliferation and migration of VSMCs. **A** Western blot analysis of MAPK signaling pathway components (ERK, JNK, p38) and their phosphorylated forms in macrophages under indicated conditions. **B**–**D** Quantitative analysis of MAPK signaling pathway-related proteins. *n* = 3. **E**–**G** M1 macrophage-related markers (TNF-α, IL-6, and IL-1β) were detected using qRT-PCR. *n* = 4. **H**, **I** Percentage of EdU-positive cells were analyzed to determine the proliferative activity of VSMCs. *n* = 3. Scale bar = 100 μm. **J**, **K** Transwell assay to detect the migration ability of VSMCs after co-culture. *n* = 3. Scale bar = 100 μm. Data are presented as mean ± SD. For in vitro experiments in this study were all repeated for at least three times. Within each independent experiment, four technical replicates (wells) were set for the Transwell migration, Edu proliferation assays and qPCR analysis; the mean values of these technical replicates were used for statistical calculations. Statistical analysis was performed using One-way ANOVA followed by Tukey’s test for (**B**–**D**), (**E**–**G**), (**I**) and (**K**). *p < 0.05, **p < 0.01, ***p < 0.001 and ****p < 0.0001

KEGG pathway enrichment analysis revealed a significant association between the MAPK signaling pathway and GPR132-mediated promotion of macrophage M1 polarization. Western blot analysis demonstrated that GPR132-OE markedly enhanced the phosphorylation levels of ERK, p38, and JNK during M1 polarization, with the most pronounced effect observed for p38 (Fig. 7A). Quantification of phosphorylation levels corroborated the results (Fig. 7B–D). These findings collectively implicate the p38/MAPK axis as a critical downstream effector of GPR132-mediated M1 polarization. To test this hypothesis, the p38/MAPK inhibitor SB203580 was used. Based on previous studies, cells were treated with 20 µM SB203580 for 1 h at 37 °C prior to stimulation [23]. The results indicated that, compared to the OE-Vector + LPS group, GPR132-overexpressing macrophages (OE + LPS) secreted significantly higher levels of inflammatory cytokines (TNF-α, IL-6, and IL-1β). This enhanced secretion was substantially attenuated upon SB203580 treatment (Fig. 7E–G), implying that GPR132 promotes M1 polarization primarily through activation of the p38/MAPK pathway. Additionally, in an indirect co-culture model, EdU proliferation assays and Transwell migration experiments showed that the increased proliferation and migration of VSMCs induced by GPR132-OE M1 macrophages were significantly suppressed by SB203580 (Fig. 7H–K). Thus, the results demonstrate that inhibition of p38/MAPK signaling using SB203580 effectively suppresses GPR132-induced M1 macrophage polarization and subsequently reduces its stimulatory effects on VSMCs proliferation and migration.

### GPR132 antagonist inhibits M1 polarization of macrophages, thereby reducing proliferation and migration of VSMCs

**Fig. 8:**
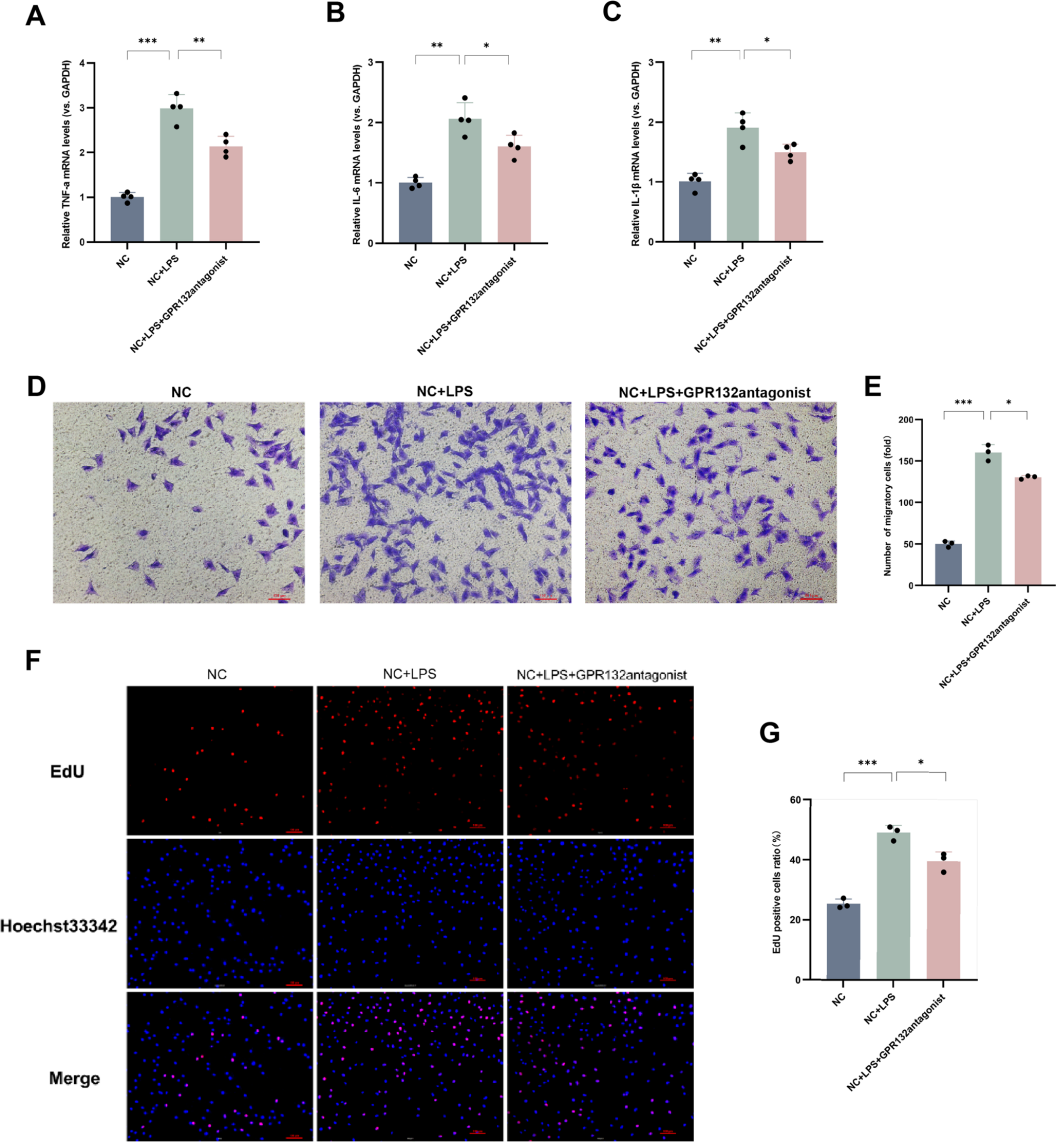
Effects of GPR132 antagonist treatment on M1 polarization of macrophages and proliferation and migration of VSMCs. **A**–**C** M1 macrophage-associated markers (TNF-α, IL-6, and IL-1β) were detected using qRT-PCR. *n* = 4. **D**, **E** Transwell assay was used to detect the migration ability of VSMCs after co-culture. *n* = 3. Scale = 100 μm. **F**, **G** The percentage of EdU positive cells was analyzed to determine the proliferative activity of VSMCs. *n* = 3. Scale = 100 μm. Data are presented as mean ± SD. For in vitro experiments in this study were all repeated for at least three times. Within each independent experiment, four technical replicates (wells) were set for the Transwell migration, Edu proliferation assays and qPCR analysis; the mean values of these technical replicates were used for statistical calculations. Statistical analysis was performed using One-way ANOVA followed by Tukey’s test for (**E**) and (**G**). *p < 0.05, **p < 0.01, ***p < 0.001

To further investigate the therapeutic potential of targeting GPR132, RAW264.7 cells were first polarized toward an M1 phenotype via LPS stimulation. Following this, macrophages were treated with 10 µM GPR132 antagonist for 3 h at 37 °C to inhibit GPR132 activity. Compared with untreated M1 macrophages (NC + LPS group), treatment with the GPR132 antagonist significantly reduced the secretion of pro‑inflammatory cytokines (Fig. 8A–C), indicating that GPR132 blockade attenuates the inflammatory response in M1 macrophages. Furthermore, using a previously established macrophage–VSMC co‑culture model, Transwell migration assays revealed that the chemotactic migration of VSMCs was markedly decreased when exposed to conditioned medium from antagonist‑treated M1 macrophages relative to the NC + LPS group (Fig. 8D–E). EdU proliferation assays demonstrated that supernatant from GPR132‑antagonist‑treated macrophages also suppressed VSMCs proliferation (Fig. 8F and G).

## Discussion

AS remains the leading cause of cardiovascular disease-related mortality. Although anti-inflammatory therapies have advanced considerably, a significant number of patients continue to experience adverse cardiovascular events [24]. Thus, identifying novel therapeutic targets for AS is critically important. This study explores the role and mechanism of GPR132, a G-protein-coupled receptor, in AS. It is demonstrated that GPR132 primarily enhances M1 polarization of macrophages via the p38/MAPK signaling pathway, leading to increased secretion of proinflammatory cytokines. The microenvironment of vascular endothelial injury was simulated by treating VSMCs with conditioned medium derived from macrophage supernatant. The research indicates that GPR132 promotes VSMCs proliferation and migration by regulating macrophage polarization. Both in vitro and in vivo experiments indicated that GPR132 antagonism attenuates AS lesion progression and reduces inflammatory responses in ApoE^−/−^ mice.

Macrophage polarization (proinflammatory M1 phenotype and anti-inflammatory M2 phenotype) plays a crucial role in the progression of AS and plaque stability [25]. M1 macrophages sustain local inflammation through the secretion of cytokines such as TNF-α, IL-6, and IL-1β, promoting monocyte infiltration and endothelial activation. In this polarized state, macrophages exhibit impaired lipid phagocytosis, contributing to plaque formation and AS advancement [26, 27]. Elevated IL-6 levels from M1 macrophages in advanced AS are associated with poor prognosis, including unstable angina and myocardial infarction [28]. The regulatory roles of G protein-coupled receptors (GPCRs) in macrophage polarization have been increasingly recognized. For instance, CCR1 has recently been shown to drive M1 polarization via KAT2A-mediated STAT1 succinylation, reinforcing the pro-inflammatory metabolic switch in macrophages [29]. CCR1 deficiency activates the PPARγ signaling pathway, promoting M2 polarization of macrophages, thereby alleviating chronic inflammatory diseases [30]. CCR2 facilitates M1 polarization through the MAPK/NF-κB pathway, contributing to inflammatory bowel diseases [31]. Additionally, CXCR7 is expressed on M1 macrophages and promotes polarization via ERK1/2 activation [32]. GPR132 is highly expressed in macrophages and is upregulated in human atherosclerotic plaques and murine AS models [33]. Previous studies suggest that GPR132 positions macrophages within inflammatory niches to drive M1 polarization [20]. GPR132 deficiency reduces M1 macrophage abundance, mitigates tissue inflammation, and promotes M2 polarization, though the underlying mechanisms remain unclear [34]. To investigate how GPR132 influences macrophage polarization, transcriptomic data from RAW264.7 and M1 macrophages from the GEO database were analyzed, confirming GPR132 upregulation in M1 macrophages. Under LPS stimulation, GPR132-overexpressing macrophages showed enhanced M1 polarization and increased inflammatory cytokine secretion (Fig. 3B–G). RNA sequencing of GPR132-overexpressing M1 macrophages revealed significant enrichment of the MAPK pathway, with notable p38/MAPK activation—a finding corroborated by inhibition with SB203580. As a GPCR family member, GPR132 likely activates p38/MAPK through Gα proteins. Similar to GPR75, which activates RAS/MAPK via Gαq/11, it is hypothesized that GPR132 initiates the PLC–PKC cascade through Gαq/11, leading to p38 phosphorylation [35]. Proton-sensing GPCR studies confirm GPR132 coupling to Gαq, triggering PLC-mediated PIP₂ hydrolysis to DAG, which activates PKC and downstream p38—consistent with the proinflammatory phenotype observed [36]. Alternatively, Gi protein signaling may also contribute: ligands such as 9(S)-HODE activate GPR132, inhibiting adenylate cyclase via Gi, reducing cAMP, and alleviating repression of p38 upstream kinases. Giβγ subunits may further directly activate kinase cascades promoting p38 phosphorylation and M1 polarization [37]. Thus, GPR132 likely activates p38/MAPK through both Gαq/11–PLC–PKC and Gi–cAMP–PKA pathways.

GPR132 is implicated in diverse biological processes, including tumor cell proliferation, invasion, breast cancer metastasis, and immune regulation [38, 39]. For example, the lactate–GPR132 axis facilitates breast cancer metastasis by enhancing tumor–macrophage crosstalk [40]. In the brain, acidic conditions promote GPR132-mediated astrocyte migration, contributing to local inflammation and fibrosis [41]. Furthermore, GPR132 binds lactate with high affinity and has been implicated in cancer and diabetes. In ovarian cancer, lactate activates GPR132 to promote macrophage infiltration and immunosuppression [42]. As a receptor for oxidized fatty acids, pharmacological modulation of GPR132 may reduce vascular risk in diabetics and ameliorate AS [43]. Interestingly, L-lactic acid was reported to activate GPR132–PKA/AMPKα1 signaling, inhibiting M1 polarization and improving insulin resistance [44]. Recent studies also implicate GPR132 in macrophage senescence: lactate activates GPR132 in diabetic models, inducing Src phosphorylation and senescence, thereby promoting foam cell formation and AS progression [45]. Additionally, GPR132 participates in inflammation regulation. Upon activation by lactate molecules, GPR132 modulates the expression of downstream inflammatory factors through signaling pathways such as PI3K/AKT/CREB, thereby contributing to immune regulatory processes [46]. In advanced AS, 9-Hydroxyoctadecadienoic acid (9-HODE) activates GPR132, increasing plaque instability [47]. These findings suggest that GPR132 may play a pivotal role in the progression of AS. In the aforementioned studies, enrichment of GPR132 expression within atherosclerotic plaques was observed in both human and mouse models (Fig. 3). This likely stems from its activation by abundant specific ligands within the local pathological microenvironment. Its endogenous ligands, including lactate and oxidized fatty acids such as 9-HODE, are markedly elevated in atherosclerotic lesions [45, 47]. Lactate accumulates due to heightened glycolytic activity of inflammatory cells, including macrophages, within hypoxic plaques [48]. Concurrently, oxidative stress in the vascular wall promotes lipid peroxidation, leading to increased levels of oxidized phospholipids and fatty acids like 9-HODE [49]. These ligands can specifically activate GPR132 signaling. For instance, lactate has been shown to engage the GPR132-Src axis, promoting macrophage senescence and foam cell formation, while 9-HODE acts as a potent agonist enhancing local inflammation [45, 47]. Thus, GPR132 agonists in atherosclerotic plaque lesions provide sustained stimulation for receptor activation and persistent expression. This ligand-driven activation likely underpins its functional role in promoting the pro-inflammatory M1 macrophage polarization observed in this study.

VSMCs exhibit phenotypic plasticity in response to local cues such as growth factors, inflammatory mediators, and intercellular signals. Excessive VSMC proliferation and migration accelerate AS [7]. To examine how GPR132 influences AS development, a macrophage–VSMCs co-culture model was established mimicking the AS vascular microenvironment (Fig. 5A). Using EdU and Transwell assays, it was found that conditioned medium from GPR132-overexpressing macrophages markedly enhanced VSMCs proliferation and migration. It is propose that GPR132 promotes M1 polarization, exacerbating plaque instability and inflammation, which in turn alters VSMCs phenotype and accelerates AS. Although Yes-associated protein was reported to suppress GPR132 expression in VSMCs and inhibit their proliferation, the mechanism by which GPR132 influences VSMC migration warrants further investigation [50]. Collectively, the results identify GPR132 as a promising therapeutic target in AS. It promotes AS progression by driving M1 macrophage polarization via the p38/MAPK pathway, enhancing inflammation, and stimulating VSMCs proliferation and migration.

GPCR antagonists show therapeutic potential in diabetes and cardiovascular disease. Diabetes is a major AS risk factor. The selective GPR132 antagonist NOX-6-18 inhibits lipid-mediated GPR132 activation, reduces cytokine release, alleviates pancreatic inflammation, and improves diabetic phenotypes [51]. Similarly, AZD3965, a lactate inhibitor, indirectly suppresses GPR132 expression and improves immune inflammation [21]. Furthermore, it can be inferred that lactate inhibitors may mitigate the progression of AS in diabetic mice by inhibiting the GPR132-Src signaling pathway [45]. These data suggest that GPR132 antagonists may play a significant role in the treatment of AS. In this study, it is shown that GPR132 antagonism significantly attenuates AS progression by suppressing M1 polarization, reducing vascular inflammation, and reducing VSMCs proliferation and migration. Notably, no toxicity was observed in the liver, kidney, lung, or spleen, indicating a favorable safety profile. GPR132 antagonists reduce plasma TNF-α and IL-1β levels without affecting TC, TG, LDL-C, or HDL-C levels. These data indicate that GPR132 antagonists possess potent anti-inflammatory effects but fail to lower lipid profiles. Compared to existing statins, GPR132 antagonists may prioritize anti-inflammatory effects over lipid metabolism regulation, potentially making them more suitable for inflammation-dominant AS subtypes. However, they cannot improve lipid metabolism. This suggests potential synergistic effects when combined with statins.

## Limitations

Several limitations of the present study warrant consideration. First, while the in vivo data demonstrate a therapeutic benefit of GPR132 antagonism, the experiments were conducted exclusively in male ApoE^−/−^ mice. Given that immune cell functions and atherosclerotic lesion progression are known to differ substantially between males and females, the translational relevance of these findings to female subjects remains to be determined. Second, although pharmacological antagonism of GPR132 was employed, the administration of a single fixed dose (2 mg/kg) without dose‑escalation or long‑term toxicokinetic evaluation precludes a comprehensive assessment of the therapeutic window and chronic safety profile of this compound. Third, while the mechanistic studies pinpoint the p38/MAPK axis as a critical downstream effector in vitro, the specific contribution of this signaling module to the anti‑atherogenic effects observed in vivo has not been formally validated using macrophage‑specific Gpr132 conditional knockout models or in vivo p38 inhibition. Fourth, although it is speculate that Gαq/11 and Gi pathways couple GPR132 to p38 phosphorylation, the exact G‑protein subtypes and the upstream scaffolding proteins involved in this relay remain unidentified. Fifth, the analyses of macrophage polarization states and GPR132 expression patterns rely heavily on publicly available external datasets (GEO) and established cell lines; while these are rigorously controlled, they cannot fully capture the heterogeneity and complex microenvironmental cues present within native human or murine atherosclerotic plaques. Collectively, these caveats do not diminish the core conclusions but underscore the necessity for further mechanistic dissection and preclinical optimization to facilitate eventual clinical translation.

## Conclusion

**Fig. 9:**
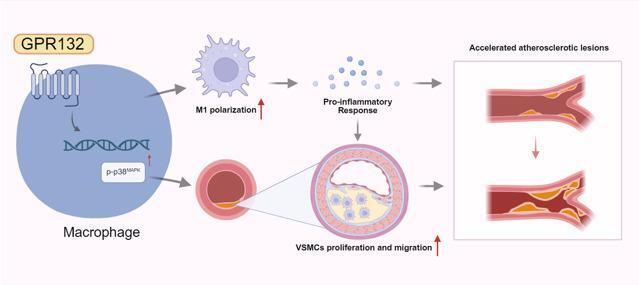
Based on the present data, it is proposed that GPR132 activates the p38/MAPK signaling pathway to promote M1 macrophage polarization, increase proinflammatory factor secretion, exacerbate the subendothelial inflammatory microenvironment, and thereby enhance VSMCs proliferation and migration. These dual effects collectively accelerate atherosclerotic progression

This study demonstrates that GPR132 promotes M1 polarization of macrophages through the p38/MAPK pathway, increases proinflammatory cytokine secretion, and enhances the proliferation and migration capacity of VSMCs. This may represent a potential mechanism for exacerbating atherosclerotic progression (Fig. 9). Pharmacological antagonism of GPR132 attenuates inflammatory responses and effectively reduces atherosclerotic plaque area in ApoE^−/−^ mice without affecting lipid metabolism. Future investigations exploring combined therapy with lipid-lowering drugs may offer novel therapeutic strategies for AS.

## Materials and methods

### Animals

All animals were maintained according to the guidelines outlined in the Guide for the Care and Use of Laboratory Animals. Apolipoprotein E-deficient (ApoE^−/−^) mice (male, 6 weeks old) and wild-type (WT) C57BL/6J mice (male, 6 weeks old) were purchased from Wuhan Savier Biotechnology Co., Ltd. (Wuhan, China). Mice were housed under specific conditions in a controlled environment (temperature: 22 ± 1 °C; humidity: 55 ± 5%; 12 h light/dark cycle) with free access to water and diet. All animal procedures were approved by the Institutional Animal Care and Use Committee of Tongji Medical College, Huazhong University of Science and Technology (Approval No. 2025021). After one week of acclimatization, mice were randomly assigned to three experimental groups (*n* = 6 per group): Control (CON): WT mice fed a standard chow diet (10% kcal fat). Model (MOD): ApoE^−/−^ mice were fed a high-fat diet (HFD, D12108C, Medicience, China), in which fat, carbohydrates, and protein accounted for 40%, 40%, and 20% of total calories, respectively, and the cholesterol content was 1.25%. Treatment (MOD/antagonist): ApoE^−/−^ mice fed the same HFD and administered a GPR132 antagonist (2 mg/kg, MedChemExpress) via intraperitoneal injection three times per week from weeks 8–12 (Supplementary Fig. S1). Body weight and food intake were monitored weekly. At the end of the 12-week experimental period, mice were fasted overnight, anesthetized with sodium pentobarbital (50 mg/kg, i.p.), and euthanized by cervical dislocation. Blood was collected via cardiac puncture into heparinized tubes, centrifuged at 3000×g for 15 min at 4 °C to obtain plasma, and stored at − 80 °C until analysis. The entire aorta and heart were carefully dissected, cleaned of perivascular adipose tissue, and either fixed in 4% paraformaldehyde for histological analysis or embedded in optimal cutting temperature (OCT) compound for frozen sections. Additional organs (liver, spleen, lung, kidney) were collected for histopathological evaluation.

### Reagents and antibodies

Cell culture medium (DMEM) was purchased from Cytiva (Massachusetts, USA), and fetal bovine serum (FBS) was purchased from Every Green (Wuhan, China). PE anti-mouse CD86 Antibody was purchased from Biolegend (San Diego, USA). SB203580 and GPR132 antagonist were purchased from MedChemExpress (New Jersey, USA). Antibodies to GPR132, β-actin, ERK, p-ERK, JNK, p-JNK, p38, and p-p38 were purchased from Affinity (Melbourne, Australia). Antibody to iNOS was purchased from Proteintech (Wuhan, China). Trizol was purchased from Cowin (Beijing, China). Edu cell proliferation kit was purchased from BeyoClick (Shanghai, China). Crystal violet aqueous solution was purchased from Solarbio (Beijing, China).

### Generation of nitric oxide

RAW264.7 cells were cultured overnight at a density of 1 × 10^4^ cells/well in a 96-well plate. Subsequently, cells were exposed to different concentrations of lipopolysaccharide (LPS, 100, 250, 500, 1000, 1500 ng/ml) for 24 h. Fifty microliters of supernatant from each group were collected. Fifty microliters of Griess Reagent I and Griess Reagent II solutions were added to each sample. The absorbance values of each group were measured at 540 nm using an enzyme-linked immunosorbent assay (ELISA) reader. A standard curve was generated by linear fitting the absorbance values of the standard samples. The NO content in the supernatant could be determined based on the standard curve.

### Cell culture and polarization stimulation

The murine aortic vascular smooth muscle cell line MOVAS and the murine macrophage cell line RAW264.7 were purchased from Pulitzer Life Sciences (Wuhan, China) in DMEM medium containing 10% fetal bovine serum (FBS) and 1% penicillin/streptomycin. Both cell lines were cultured in T25 cell culture flasks and placed in a constant chamber at 37 °C with 5% CO_2_. The MOVAS cell line was cultured to 4–5 generations for use in the experiments. When RAW264.7 reached 80% confluence, cells were scraped, dissociated and counted, and then inoculated in 6-well plates at a concentration of 2 × 10^5^/ml. When 60% confluence was reached, cells were stimulated with 1 µg/ml pg-LPS (InvivoGen, San Diego, CA, USA) and 20 ng/ml IL-4 (Biolegend, San Diego, CA, USA) for 24 h to induce M1 and M2 macrophage polarization. The mRNA levels of proinflammatory cytokines (TNF-α, IL-1β, IL-6, and iNOS) and anti-inflammatory cytokines (IL-10 and Arg1) were detected via qPCR to validate successful induction of M1 macrophage polarization [52].

### Oil red O staining

The aorta root that attached to the heart was used for cross-sections. The collected aortic tissue was fixed in 4% paraformaldehyde for 24 h and dehydrated overnight. Then, the tissue was embedded in the embedding agent OCT to prepare frozen slices (5 μm) at − 20 °C. A saturated oil red O solution dissolved in isopropanol was mixed with distilled water at a ratio of 3:2 (v: v). After filtration, the frozen slices were stained in the diluted oil red O working solution at room temperature for 6 h. Then, the slices were rinsed in 60% isopropanol-water solution 3 times and once in running water. The slices were restrained in hematoxylin solution for 1–2 min and washed with running water. The dyed slices were sealed with neutral gelatin, photographed and recorded by a microscope system (Leica, Germany). The corrected plaque areas were calculated as follows: Area of plaques/Area of vascular × 100% in the corresponding cross section. Lesion size was measured on digital microphotographs using ImageJ software. The statistical results of the corrected plaque area were the average values of the plaque area obtained by oil red O staining of each mouse. To ensure valid comparative assessment of plaque burden across experimental groups, aortic root cross-sections were collected from a standardized anatomical location. Briefly, the upper section of the aortic root, immediately adjacent to the aortic valve leaflets (approximately at the level of the sinus of Valsalva), was identified and consistently used for sampling across all animals.

### Hematoxylin-eosin (H&E) staining

The aortic root were fixed in 4% paraformaldehyde for 24 h after being collected and dehydrated for 16 h by an automatic dehydrator. After routine embedding with paraffin in an automatic embedding machine, the tissue was cut into sections (5 μm). Then, the sections were placed in an oven (60 °C) for 1 h and dewaxed with xylene, ethanol and water. The sections were further stained with hematoxylin for 10 min and placed under running water to remove residual color. Then, 5% acetic acid was used to differentiate the cells, which were then stained with eosin. Finally, the sections were sealed with neutral gelatin after drying, and images were captured by the microscope system.

### Serum and plasma analysis

Blood samples were collected in heparinized tubes and centrifuged at 3000×g for 15 min at 4 °C to separate plasma. Serum was obtained by allowing blood to clot at room temperature for 30 min, followed by centrifugation as above. Levels of TNF-α and IL-1β were quantified using ELISA kits (R&D Systems) according to the manufacturer’s instructions. Lipid profiles (TG, T-CHO, LDL, HDL) were measured with enzymatic colorimetric assays (BioVision) on a Beckman Coulter AU5800 analyzer. All samples were assayed in triplicate technical replicates.

### Acquisition and analysis of public scRNA-seq data

Two publicly available single-cell RNA sequencing (scRNA-seq) datasets were obtained from the Gene Expression Omnibus (GEO) database. The murine atherosclerosis dataset (GSE155513) consisted of cells isolated from the arterial tissues of ApoE^−/−^ SMC-lineage tracing mice fed a Western diet for 16 weeks. The human carotid atherosclerosis dataset (GSE159677) contained cells derived from calcified atherosclerotic core plaques and patient-matched adjacent tissues from patients undergoing carotid endarterectomy. Raw gene expression matrices were processed using the R package Seurat (version 5). Stringent quality control (QC) criteria were applied to filter out low-quality cells. For the murine dataset, cells with detected gene numbers (nFeature_RNA) between 200 and 5000, and mitochondrial gene percentages (percent.mt) < 15% were retained. For the human dataset, cells with nFeature_RNA between 200 and 5000, and percent.mt < 20% were retained. Following QC, data were normalized using the NormalizeData function. The top 2000 highly variable features were identified using the FindVariableFeatures function, followed by scaling and centering with ScaleData. Principal component analysis (PCA) was performed for linear dimensionality reduction. The top 20 principal components (PCs) were selected for graph-based clustering (FindNeighbors and FindClusters with a resolution of 0.5) and non-linear dimensionality reduction using Uniform Manifold Approximation and Projection (UMAP). To further analyze macrophage phenotypes, the annotated macrophage clusters were subsetted and divided into GPR132-High and GPR132-Low groups based on the detectable expression levels of GPR132 (mouse) or GPR132 (human). Inflammatory polarization (M1 signatures) and paracrine growth factor expression profiles were subsequently evaluated using DotPlot and VlnPlot visualizations.

### Construction of GPR132 knockout macrophage cell lines

Knockout plasmid was constructed with the target amplification primers GPR132-PF as follows (gtatgtatgtgtgccctgtgtgtgtatg), GPR132-PR (ccatttgtgctgattctggatg). For transduction of RAW264.7 cells, 1 × 10^5^ cells per well were inoculated into 24-well plates. After overnight incubation, cells were co-transduced with 2 × 10^6^ TU of lentiviral vectors expressing Cas9- ha - nls and sgRNA (thus, MOI of 20), or with 2 × 10^6^ TU of a single lentiviral vector expressing CR2 sgRNA and Cas9 in the presence of 1 µg/ml polyglutamine (thus, MOI of 20). Seven days after transduction, the cells were detected by polyclonal assay followed by FACS analysis.

### Construction of macrophage cell lines overexpressing GPR132

The pCDH-CMV-MCS-CopGFP-T2A-Puro empty plasmid was used with BamHI and NotI double enzyme digestion was performed.The amplified target fragment was homologously recombined with the digested empty load. The empty plasmid and overexpression plasmid were co-transfected with lentiviral-packaged helper plasmid into HEK293T cells, and the viral fluids were collected after 48 h and 72 h of transfection, and then filtered and concentrated to obtain the empty viral fluids and overexpression viral fluids. The packaged empty and overexpressed lentiviruses were infected with RAW264.7 cells according to the MOI of 20, and fluorescence pictures were taken at 48 h of infection, and puro at a final concentration of 1ug/ml was added for drug screening, which could be detected 5–7 days after drug screening. Detection and by polyclonal detection, followed by FACS sorting.

### Reverse transcription quantitative real time PCR (RT-qPCR)

RNA was isolated using TRIzol, followed by qPCR with 1 µg of total RNA (+ gDNA wiper) and reverse transcription reactions with a HiScript II Q RT SuperMix (+ gDNA wiper) and gDNA eraser (R223-01, Vazyme, China). qRT-PCR assays were performed using SYBR Green SuperMix and StepOne Software v.2.3 (Applied Biosystems) for 40 cycles (three biological replicates). Data were analyzed using the −ΔΔCt (cycle threshold) method and normalized to the expression of the reference gene GAPDH. Primers for GPR132, TNF-α, IL-6, iNOS, IL-1β and GAPDH were purchased from Tsingke (Beijing, China) and had the following sequences. The PCR primer sequences used in RT-qPCR are provided in Supplementary Table S1.

### Flow cytometry analysis

RAW264.7 cells were seeded in 6-well plates and harvested 24 h after LPS treatment, counted and treated with CD86 antibody (GL-1, PE, 105008) in the dark for 30 min. The cells were then subjected to BD Accuri C6 flow cytometer (BD, Franklin Lakes, NJ, USA). to detect cell staining. All conditions were repeated three times.

### Western blot analysis

Total protein was extracted from RAW264.7 macrophages using RIPA lysis buffer (Beyotime, Shanghai, China) supplemented with protease and phosphatase inhibitor cocktail (Beyotime). Protein concentration was determined by BCA assay. Equal amounts of protein (20 µg per lane) were separated by 10% SDS-PAGE and transferred onto PVDF membranes (Millipore) at 300 mA for 30 min. Membranes were blocked with 3% BSA in TBST for 30 min at room temperature and then incubated overnight at 4 °C with the following primary antibodies: anti-β-actin (Proteintech, 81115-1-RR, 1:20,000), anti-ERK (Affinity, AF0155, 1:20,000), anti-phospho-ERK (p-ERK; Affinity, AF1015, 1:2,000), anti-p38 (Proteintech, 14064-1-AP, 1:4000), anti-phospho-p38 (p-p38; Proteintech, 28796-1-AP, 1:2000), anti-JNK (Proteintech, 51151-1-AP, 1:2000), and anti-phospho-JNK (p-JNK; Affinity, AF3318, 1:20,000). Following three washes with TBST, membranes were incubated with a secondary antibody for 30 min. Protein bands were visualized using Bio-Rad’s Western ECL Substrate kit and quantified using ImageJ software.

### Cell migration assay

The VSMCs were incubated in a 6-well plate at a density of 5 × 10^5^. When the cells were 95% full, the VSMCs were starved in a serum-free medium. After 12 h, the bottom of the plate was scraped with the tip of a 200-µL sterile pipette to produce a 1-mm-wide wound, and the cells were then incubated at 37 °C for 24 h. The relative migration distance of VSMCs into the scratch area was recorded and quantified using ImageJ software. Transwell migration assays were performed using a 24-well Transwell chamber (Corning, NY, USA). VSMCs (1 × 10^5^) were evenly spread into the upper chamber of transwell plates (pore size 8 μm, corning) and cultured in 100 µL serum-free medium. A mixture of 700 µL DMEM containing 20% FBS and M1 macrophage supernatant was added to the lower chamber. After 24 h of culture at 37 °C at 5% CO_2_, these cells had migrated through the membrane and were fixed with 4% formaldehyde for 20 min and 0.1% crystal violet solution for 30 min. Cells from the experimental and control groups were photographed and counted under an inverted phase contrast microscope (Olympus, Tokyo, Japan). The migrated cell population was evaluated in three random fractions under the microscope. Data are expressed as the difference in the number of VSMCs migrating compared to the corresponding control.

### Cell proliferation assay

Cell proliferation was measured with Cell Counting Kit-8 (CCK-8) proliferation assay (Abbkine, Wuhan, China) and EdU kit (Beyotime Biotechnology, Shanghai, China) according to the manufacturer’s instructions. Cells were starved overnight and 3 × 10^3^ VSMCS/well were seeded in 96-well plates for adherence. After the cells were adherent, macrophage supernatant was added. CCK-8 reagent was added to each well and the absorbance at 450 nm was measured after 1 h of incubation at 37 °C. The EdU assay was carried out according to the manufacturer’s instruction. The representative images acquired by fluorescence microscope (Olympus, Tokyo, Japan). The cell proliferative rate was calculated as the proportion of Hoechst 33,342-staining cells that incorporated EdU in 10 high-power fields per well. The results were quantified by fluorescence microscopy and analyzed by Image J software.

### Acquisition and differential expression analysis of public RNA‑seq datasets

To profile macrophage polarization-associated transcriptional changes, raw gene expression count matrices for datasets GSE183077, GSE230444, and GSE283748 were downloaded from the GEO database. These datasets were selected based on the availability of transcriptomic profiles from RAW264.7 macrophages in the M0 state and following M1 polarization. For each dataset, the raw count tables corresponding to the GPL platform used were extracted. Lowly expressed genes with a counts‑per‑million (CPM) value < 1 in all samples were filtered out to reduce background noise. To correct for potential batch effects across the three independent GEO datasets, the ComBat‑seq method implemented in the sva R package (v3.38.0) was applied, using the experimental condition (M0 vs. M1) as a covariate. Normalization and differential expression analysis were performed using the DESeq2 R package (v1.30.1) with the median‑of‑ratios method. Differential expression was defined as |log₂(fold change)| > 1 and an adjusted *P* value (Benjamini‑Hochberg false discovery rate correction) < 0.05. Principal component analysis (PCA) was performed using the prcomp function in R (v4.0.3) to confirm clustering of samples by polarization status. Heatmaps and volcano plots were generated using the pheatmap (v1.0.8) and ggplot2 (v3.3.3) R packages, respectively. All RNA‑seq bioinformatic analyses were conducted in the R statistical environment (v4.0.3).

### RNA sequencing and bioinformatics analysis

GPR132‑OE and OE‑Vector RAW264.7 macrophages were seeded in 6‑well plates at 2 × 10^5^ cells/mL. Upon reaching 60% confluence, cells were stimulated with 1 µg/mL lipopolysaccharide (LPS) for 24 h to induce M1 polarization. Following LPS stimulation, total RNA was isolated using TRIzol reagent (Invitrogen, CA, USA). RNA purity and concentration were assessed using a NanoDrop spectrophotometer and Qubit 4.0 fluorometer, while RNA integrity was evaluated on an Agilent 2100 Bioanalyzer; only samples with an RNA integrity number (RIN) ≥ 7.0 were processed for downstream applications. Polyadenylated mRNA was enriched from 3 µg of total RNA using poly‑T oligo‑attached magnetic beads (Vazyme Biotech, Nanjing, China). Fragmentation was carried out using divalent cations under elevated temperature in fragmentation buffer. First‑strand cDNA was synthesized using random hexamer primers, followed by second‑strand cDNA synthesis with buffer, dNTPs, RNase H, and DNA polymerase I. The resulting double‑stranded cDNA was subjected to end‑repair, A‑tailing, and ligation to sequencing adaptors. Size selection and purification were performed using Hieff NGS^®^ DNA Selection Beads (Yeasen Biotechnology, Shanghai, China). The final cDNA libraries were amplified by PCR with 12–15 cycles and quantified using Qubit^®^ 4.0. Library quality and fragment size distribution were verified on an Agilent 4200 TapeStation. Paired‑end sequencing (150 bp) was performed on the DNBSEQ‑T7 platform (MGI Tech, Shenzhen, China) at LingSi Biotechnology Co., Ltd. (Wuhan, China). Each experimental group contained three independent biological replicates. For data processing, raw FASTQ reads were filtered using fastp (v0.21.0) to remove adaptor sequences and low‑quality bases (Q < 20). High‑quality clean reads were aligned to the mouse reference genome (GRCm39/mm39) using HISAT2 (v2.1.0). Gene‑level read counts were quantified with StringTie (v2.1.5) and normalized to fragments per kilobase of transcript per million mapped reads (FPKM). Differential expression analysis between the OE and OE‑Vector groups was performed using DESeq2 (v1.30.1), with significantly differentially expressed genes (DEGs) defined by an absolute |log₂(fold change)| > 1 and an adjusted *p* value (padj) < 0.05.

### GO and KEGG enrichment analysis

All genes were mapped to terms in the Gene Ontology (GO) database, and the number of differentially enriched genes in each term was calculated. The GO enrichment analysis of differentially expressed genes was performed using Cluster Profiler, and the *P* value was calculated by the hypergeometric distribution method (the criterion for significant enrichment was padj0.05). The GO terms with significant enrichment of differentially expressed genes were identified to determine the main biological functions of differentially expressed genes. The KEGG pathway enrichment analysis of differentially expressed genes was performed using ClusterProfiler (v3.18.1) software, focusing on pathways significantly enriched at the padj0.05 level.

### Data and statistical analysis

The experiments were conducted a minimum of three times. GraphPad Prism 9.0 software was utilized for data analysis, and the results were displayed as means ± standard deviation for all quantitative data. Unpaired t-tests were implemented for between-group comparisons, while one-way analysis of variance (ANOVA) was used for comparisons among multiple groups. Results were marked statistically significant when *P* < 0.05. The results were visualized using DESeq2 package in R. The investigators performing the Oil Red O staining quantification, immunohistochemistry analysis, and cell counting were blinded to the experimental group allocation.

## Supplementary Information

Below is the link to the electronic supplementary material.

Supplementary Material 1

Supplementary Material 2

Supplementary Material 4

Supplementary Material 5

Supplementary Material 6

Supplementary Material 8

Supplementary Material 7

Supplementary Material 8

Supplementary Material 9

Supplementary Material 10

## Data Availability

The RNA sequencing data (GSE183077, GSE230444, GSE283748) and single-cell RNA sequencing data (GSE155513, GSE159677) used in this study were obtained from the Gene Expression Omnibus database. The raw RNA sequencing read data generated in this study have been deposited in the NCBI Sequence Read Archive (SRA) under BioProject ID [PRJNA1490462].
