# Supplementary material for: GPR132 antagonists alleviate atherosclerosis by suppressing M1 polarization and subsequent VSMCs proliferation and migration as revealed by transcriptomic profiling
Source: Inflamm Res. 2026 Aug 18;75(1):190. doi: 10.1007/s00011-026-02342-2 (PMC13481467; doi:10.1007/s00011-026-02342-2)
Supplement: Supplementary file 1 [file 11_2026_2342_MOESM9_ESM.docx]

**PCR primer sequences**

| Primer Name | Primer Sequence (5' to 3') | Base number |
| --- | --- | --- |
| GPR132(F) | TCAGGACTGGCTTGGGTCATT | 21 |
| GPR132(R) | TCCTGCTGCATTGGTAGGTT | 20 |
| GAPDH(F) | GCGACTTCAACAGCAACTCCC | 21 |
| GAPDH(R) | CACCCTGTTGCTGTAGCCGTA | 21 |
| IL-1β(F) | CACTACAGGCTCCGAGATGAACAAC | 25 |
| IL-1β(R) | TGTCGTTGCTTGGTTCTCCTTGTAC | 25 |
| IL-6(F) | CTTCTTGGGACTGATGCTGGTGAC | 24 |
| IL-6(R) | AGTGGTATCCTCTGTGAAGTCTCCTC | 26 |
| TNF-α(F) | TGTCCATTCCTGAGTTCTG | 19 |
| TNF-α(R) | GGAGGCAACAAGGTAGAG | 18 |
| iNOS(F) | TCCTCACTGGGACAGCACAGAATG | 24 |
| iNOS(R) | GTGTCATGCAAAATCTCTCCACTGCC | 26 |
| Arg1(F) | CAGAAGAATGGAAGAGTCAG | 20 |
| Arg1(R) | CAGATATGCAGGGAGTCACC | 20 |
| IL-10(F) | GCTCTTACTGACTGGCATGAG | 21 |
| IL-10(R) | CGCAGCTCTAGGAGCATGTG | 20 |
